# Focusing a round coherent beam by spatial filtering the horizontal source

**DOI:** 10.1107/S1600577520012163

**Published:** 2020-10-20

**Authors:** Eric M. Dufresne, Suresh Narayanan, Ruben Reininger, Alec R. Sandy, Larry Lurio

**Affiliations:** a Advanced Photon Source, Argonne National Laboratory, Argonne, IL 60439, USA; b Department of Physics, Northern Illinois University, DeKalb, IL, USA

**Keywords:** X-ray photon correlation spectroscopy, refractive lenses

## Abstract

This paper illustrates the use of spatial filtering with a horizontal slit near the source to enlarge the horizontal coherence in an experimental station and produce a diffraction-limited round focus at an insertion device beamline for X-ray photon correlation spectroscopy experiments. Simple expressions are provided to guide the optical layout, and wave propagation simulations confirm their applicability. The two-dimensional focusing performance of Be compound refractive lenses to produce a round diffraction-limited focus at 11 keV capable of generating a high-contrast speckle pattern of an aerogel sample is demonstrated. The coherent scattering patterns have comparable speckle sizes in both horizontal and vertical directions. The focal spot sizes are consistent with hybrid ray-tracing calculations. Producing a two-dimensional focus on the sample can be helpful to resolve speckle patterns with modern pixel array detectors with high visibility. This scheme has now been in use since 2019 for the 8-ID beamline at the Advanced Photon Source, sharing the undulator beam with two separate beamlines, 8-ID-E and 8-ID-I at 7.35 keV, with increased partially coherent flux, reduced horizontal spot sizes on samples, and good speckle contrast.

## Abstract 2

The use of spatial filtering with a horizontal slit near the source to enlarge the horizontal coherence in an experimental station and produce a diffraction-limited round focus at an insertion device beamline for X-ray photon correlation spectroscopy experiments is illustrated. This scheme has now been in use since 2019 for the 8-ID beamline at the Advanced Photon Source, sharing the undulator beam with two separate beamlines, 8-ID-E and 8-ID-I at 7.35 keV, with increased partially coherent flux, reduced horizontal spot sizes on samples, and good speckle contrast.

## 1. Introduction   

Synchrotron light sources produce partially coherent light that has enabled new techniques such as X-ray photon correlation spectroscopy (XPCS) to flourish into mature material characterization methods (Sutton, 2008 ▸; Livet & Sutton, 2012 ▸; Sinha *et al.*, 2014 ▸; Sandy *et al.*, 2018 ▸). Due to the large mismatch between the horizontal and vertical source size in third-generation synchrotron sources, which is typically a factor of 20 larger in the horizontal direction, the coherence length is a factor of 20 smaller in the horizontal than in the vertical direction. As a result, beamlines specializing in XPCS often focus the vertical coherent fan to match the smaller horizontal coherence length on the sample (Sandy *et al.*, 2010 ▸; Chsuhkin, 2020 ▸).

Beamline 8-ID currently focuses the vertical coherent flux with a 1D parabolic Be lens. It is done at both branches, the 8-ID-I main line which specializes in small-angle XPCS, and the side branch 8-ID-E which focuses on wide-angle XPCS. One typically focuses the 125 µm vertical coherent beam on the sample to a few µm. The horizontal coherence is typically spatially filtered by a horizontal aperture set between 10 and 20 µm, placed 0.2 to 1.5 m before the sample, with the ranges given to show the typical openings and distances for the E and I branch, respectively. This optical scheme leads to different beam sizes on the sample and as a result different speckle sizes on the detector (Sutton, 2008 ▸).

For some experiments on beamline 8-ID, one would like to use a 2D focusing compound refractive lens (CRL) with similar focal length as the existing 1-ID lens to produce a beam that better matches the micro-structure of materials. Focusing on a small spot size also produces larger speckle sizes which can be better suited for a large-pixel pixel array detector (PAD) with typical pixel size of 0.1 mm. One would like to illuminate the sample with a 2D focused coherent beam, so, to achieve this, naively, one would reduce the horizontal beam size in the upstream hutch 8-ID-A with a slit to illuminate the lens coherently.

By choosing an appropriately small size of the horizontal slit, the diffracted beam will fill the lens with transversely coherent light. The flux will be reduced, but with the proper slit the optics will produce a diffraction-limited spot size.

We note that using a slit as a spatial filter is a well known technique at synchrotrons. It is used on the X-ray Nanoprobe for example at the APS (Winarski *et al.*, 2012 ▸). On that beamline, a horizontal mirror focuses the horizontal fan of the source onto a beam-defining aperture that filters a coherent fan of light for the X-ray optics. This aperture produces a secondary horizontal source and should be placed as close as possible to the source. It enables the production of round beams on the sample after focusing with a short focal length zone plate.

Experimenters at the Petra III beamline P10 routinely use two-dimensionally focused beams in coherent diffraction experiments (Zozulya *et al.*, 2012 ▸). Due to the very small horizontal source size at this facility, one can focus horizontally 50–100 µm beams coherently to a few µm on the sample without closing any near front-end horizontal slit. This type of source spatial filtering allows one to deliver round focused coherent beams at existing synchrotrons with modest horizontal transverse coherent lengths (10 µm or below).

Recently, several authors have modeled the propagation of coherence through optical systems including refractive lenses (Singer & Vartanyants, 2014 ▸; Schroer & Falkenberg, 2014 ▸). The first paper provides an analytical treatment which includes a slit placed just upstream of the lens and shows its impact to control the focal coherence length and spot size (Singer & Vartanyants, 2014 ▸). This can be applied to the vertical focusing direction in this paper, although it approximates the slit by a Gaussian aperture. Since it does not include a treatment adding the horizontal secondary slit, we choose to simulate our results with advanced ray-tracing simulations.

Several ray-tracing approaches have been developed which include the propagation of coherence through optical elements from a synchrotron (Chubar *et al.*, 2013 ▸; Shi *et al.*, 2014 ▸; Wiegart *et al.*, 2019 ▸). The work of Wiegart *et al.* (2019 ▸) is noteworthy for comparing measured coherent diffraction patterns from a thin grating sample illuminated by the 11-ID beamline of the National Synchrotron Light Source with simulations from the *Synchrotron Radiation Workshop* software (Chubar *et al.*, 2013 ▸). The paper also describes the optical scheme of the beamline which focuses the beam in two dimensions with a vertical transfocator upstream in the optics hutch and a horizontal kineform lens in the experimental hutch (Wiegart *et al.*, 2019 ▸).

In this paper we present a demonstration of 2D focusing with a long-focal-length Be lens to produce a round beam. We report the speckle size and contrast measured with focused coherent light. We present also optical simulations performed with a hybrid coherent diffraction module (Shi *et al.*, 2014 ▸).

## 2. Optical modeling   

**Figure 1:**
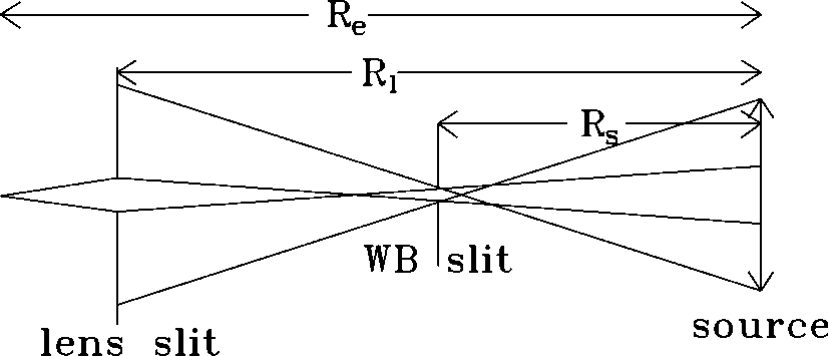
The horizontal rays collimation geometry.

The basic concept is to use a slit as a coherence filter in the horizontal direction so that its diffraction pattern in the lens plane matches the vertical transverse coherence length at the lens location. The lens can then be coherently illuminated by placing just upstream of the lens a square aperture with an opening equal to the vertical transverse coherence length 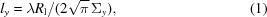
where Σ_*y*_ is the root-mean-square (RMS) vertical source size, and *R*_l_ is the distance source–lens (Dierker, 1997 ▸; Dufresne *et al.*, 2002 ▸). The distance *R*_l_ is shown in Fig. 1 ▸. The factor 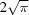 comes from the definition of the transverse coherence length for a Gaussian incoherent source from the Van Cittert–Zernicke theorem (Goodman, 1985 ▸; Dierker, 1997 ▸).

Then, a horizontal slit placed upstream of the lens with an opening Δ will produce a diffraction pattern with a full width at half-maximum (FWHM) approximately equal to 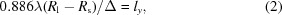
where *R*_s_ is the distance from the source to the upstream horizontal slit. The factor 0.886 comes from the FWHM of the Fraunhofer diffraction pattern of a 1D aperture with opening Δ. If one assumes that the horizontal source is spatially uniform at the white-beam slit and incoherent, the horizontal transverse coherence length at the lens is 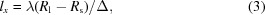
using the definition found in Goodman (1985 ▸).

Assuming that the undulator source is Gaussian in spatial and angular emission, the RMS horizontal beam size at a distance *R*_s_ from the source is approximately equal to 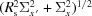, where 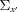 is the source horizontal divergence and Σ_*x*_ the source size. The X-ray source divergence is 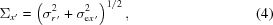
where 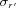 = (0.5λ/*L*)^1/2^ is the single electron source divergence through an undulator of length *L*, and 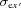 is the electron bunch RMS divergence. The X-ray vertical source size, 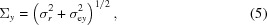
is the convolution of the single electron source size σ_*r*_ = (2λ*L*)^1/2^/(2π) and the RMS electron beam size σ_e*y*_, so there is a 6% correction for hard X-rays of 1 Å for a *L* = 2.4 m-long undulator (Elleaume, 2002 ▸). A similar equation to equation (5) can be written in the horizontal direction for Σ_*x*_, but the energy dependence is negligible due to the much larger electron beam size σ_e*x*_, thus Σ_*x*_ ≃ σ_e*x*_.

We note that the flux through the aperture is proportional to Δ/*R*_s_, when 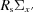 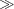 Σ_*x*_. The horizontal beam size at the APS becomes dominated by the horizontal divergence for distances larger than 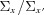 ≃ 22 m (see Table 1 ▸), which is upstream of the front-end.

Putting equation (1) into equation (2), one finds 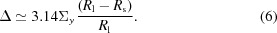
It is fairly independent of wavelength if one neglects the small energy dependence of the source size. The throughput from slitting the horizontal can be approximated by 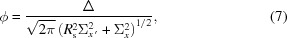
where ϕ is the partially coherent flux transmitted through the slit, assuming the slit opening Δ is much smaller than the beam profile at the slit position. By inspection of equation (7), Δ is largest when *R*_s_ = 0, and the beam size is also the smallest, thus the defining slit should be as close as possible to the source to optimize the flux. At the APS, white-beam slits are located after the front-end, but at some synchrotrons they are accessible before the front-end (Wiegart *et al.*, 2019 ▸). Reducing the horizontal beam also reduces the power on X-ray optics downstream of the slit.

At the APS, the RMS horizontal and vertical electron beam size are, respectively, σ_e*x*_ = 0.275 mm and σ_e*y*_ ≃ 0.010 mm, so they differ by a factor of 27. For a ratio *R*_s_/*R*_l_  ≃ 1/2, the small opening Δ will reduce the source divergence such that the main contribution is the pinhole camera of the source through the aperture 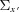 ≃ Σ_*x*_/*R*_s_, thus for *R*_s_ = 27 m, 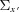 = 10 µrad. We note that, from equation (4), 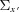 ≃ 13.5 µrad at 11 keV.

The RMS beam size at the lens position will be the convolution of the diffraction pattern of the slit Δ and beam size propagated through the aperture. Assuming that the source irradiance is both Gaussian in space and angular emission, one can show that the RMS beam profile at the lens should be written as 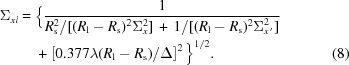
The first term is derived in Appendix *A* [App appa] and comes from the pinhole camera image of the horizontal source with finite source size and divergence. The second term on the sum in equation (8) is the width of the diffraction pattern of the slit with opening Δ. We note that an exact treatment is discussed for a bending magnet pinhole camera diagnostic (Yang *et al.*, 2017 ▸) including Fresnel diffraction. This is not needed as we are in the far-field of the slit.

Looking back at Fig. 1 ▸, one can show that the two slits form a collimator with collimation angle 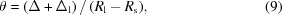
where Δ_l_ is the slit opening in front of the lens. This collimator limits the size of the source to a perceived horizontal size 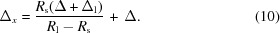
Using values found in Table 1 ▸ for the distances, and white-beam slit opening Δ, while using Δ_l_ = 0.125 mm, one finds Δ_*x*_ = 0.138 mm. Using equation (3), this collimation increases the source transverse coherence length at the white-beam slit to 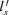 = λ*R*_s_/Δ_*x*_ = 23 µm for values defined in Table 1 ▸ and used in this paragraph. In this example, 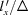 = 71.5%, which is an improvement from the original ratio *l*_*x*_/Δ ≃ 10% in Table 1 ▸.

The focal length of a parabolic compound refractive lens in the thin lens approximation is given by 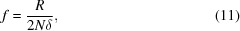
where *R* is the radius of curvature of a lenslet, *N* is the number of lenslets, and δ is the index of refraction decrement (Snigirev *et al.*, 1996 ▸; Lengeler *et al.*, 2002 ▸). When the lens can view the full source, if the source–lens distance *R*_l_ 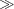 *f*, we expect the RMS focal spot size to be a demagnified image of the source with σ_i_ ≃ *f*Σ_s_/*R*_l_, where Σ_s_ is the RMS source size.

If we use a small white-beam slit opening instead, then a rough estimate of the focal spot size is 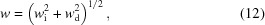
where *w*_d_ = 0.886λ(*R*_e_ − *R*_l_)/Δ_*l*_, which is the FWHM of the diffraction pattern of the lens slit aperture at the focal distance *R*_e_ measured from the source. In equation (12), the demagnified source image width, 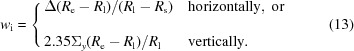
In equation (13), we use the white-beam slit opening as the source for the demagnified horizontal source width. For values found in Table 1 ▸, we find the FWHM horizontal spot size *w* = 2.6 µm.

We define the depth of focus as the distance Δ*z* from the best focal plane such that the FWHM of the beam is 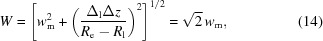
where *w*_m_ is the computed focal spot in equation (12). One finds 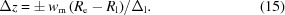
Note that *w*_m_ depends on Δ_l_, the slit opening before the lens. Using the vertical minimum focus, *w*_m_ = 2.0 µm, the vertical depth of focus is Δ_*z*_ = 3.7 cm.

## 3. X-ray optical simulations   

**Figure 2:**
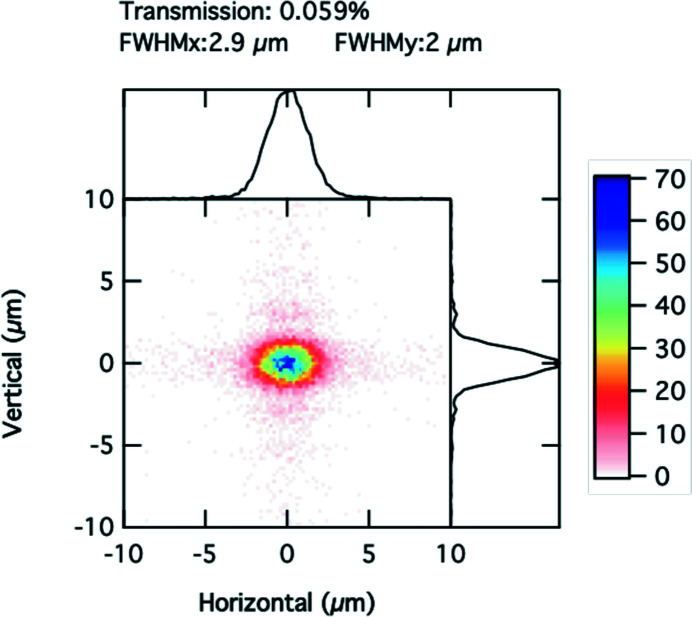
Simulated focal spot profile in 8-ID-I with a white-beam slit opening of 32 µm, 2.29 m from the lens. The central part of the figure shows the intensity distribution. The two graphs above and at the right in the figure show the cumulative intensity in each direction. The color scale displays the intensity.

X-ray optical simulations were performed with *Shadow* using the Hybrid method reported by Shi *et al.* (2014 ▸). The X-ray energy was set to 11.0 keV so that the focal distances would match the experimental setup described in Table 1 ▸. The two-dimensional aperture set to 0.125 mm × 0.125 mm is placed 59 cm before the lens. Fig. 2 ▸ shows the simulation results in the plane with the best vertical focus of a 2D Be lens. The focal spot size in the horizontal and vertical direction is shown in the plot legend as well as the transmission of the optical system of 0.059%. We note that clear diffraction effects are noticeable in the vertical direction due to the fact that the transverse coherence length vertically is larger than Δ_l_ = 0.125 mm.

Equation (12) provides a reasonable estimate of the horizontal spot size (in the simulations here 2.9 µm) to be 2.6 µm as discussed earlier. Using equation (12), and data from Table 1 ▸, we find the FWHM of the vertical spot size *w* = 2 µm, in excellent agreement with the simulations.

**Figure 3:**
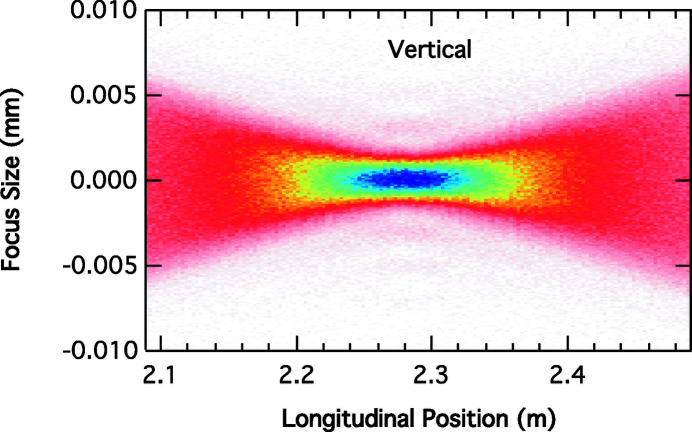
Simulated vertical focal spot profile in 8-ID-I as a function of the distance from the lens with a white-beam slit opening of 32 µm.

Fig. 3 ▸ shows the vertical spot size as a function of the longitudinal distance from the lens. The calculated distance from the lens using the thin lens formula is 2.29 m and corresponds to the vertical focal distance in the simulations. In these simulations, the source is assumed to be Gaussian and centered on the center of the 8-ID straight section.

**Figure 4:**
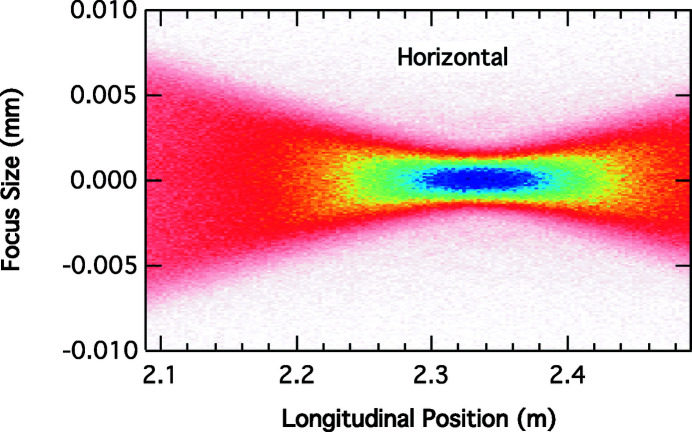
Simulated horizontal focal spot profile in 8-ID-I as a function of the distance from the lens with a white-beam slit opening of 32 µm.

Fig. 4 ▸ shows the horizontal spot size as a function of the longitudinal distance from the lens. The horizontal focal plane is about 5 cm downstream from the vertical focal position. Using the thin lens formula with the horizontal source position at the white-beam slit location, we find the image distance from the lens should be 2.344 m, consistent with the simulations.

**Figure 5:**
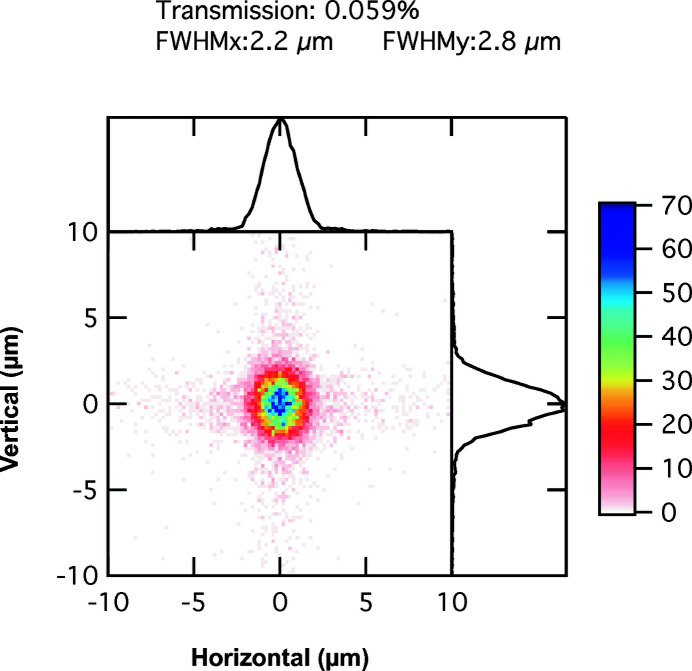
Simulated focal spot profile in 8-ID-I 2.34 m from the lens with a white-beam slit opening of 32 µm showing the smallest horizontal spot size.

Fig. 5 ▸ shows the focal spot at the best horizontal focus in Fig. 4 ▸. Because the horizontal and vertical source points are not in the same plane, it is not possible to optimize both spot sizes simultaneously. The focal area, *i.e.* the product of the vertical and horizontal FWHM beam sizes, only varies by 6% between the two planes defined in Figs. 2 ▸ and 5 ▸. The distance between the horizontal and vertical best focus, here 5 cm, compares with the vertical depth of focus of 3.7 cm computed in equation (15), but both distances are much larger than a typical sample thickness (∼3 mm) used in our XPCS experiments, thus the astigmatic focus does not cause significant beam waist changes over the illuminated length. An experimenter has the choice to explore beam profile extremes shown in Figs. 2 ▸ and 5 ▸ by moving the sample along the beam direction.

**Figure 6:**
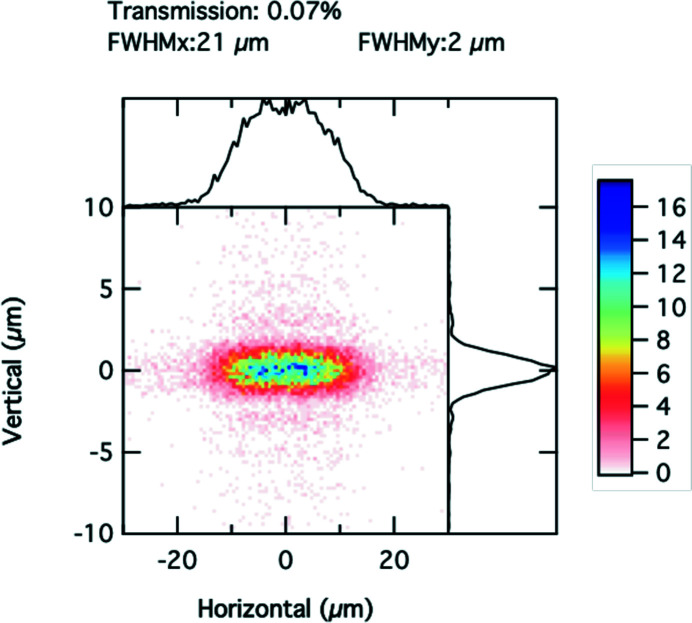
Simulated focal spot profile in 8-ID-I, using vertical focusing only with a wide-open white-beam slit opening.

Simulations were also performed using a 1D, vertically focusing Be CRL. Fig. 6 ▸ shows the focal spot profile, with a horizontal coherence slit set to 20 µm, placed 88 cm after the lens. The vertical focal spot has also a FWHM of 2.0 µm, while the horizontal profile is slightly broadened by Fraunhofer diffraction to a FWHM of 21 µm. The transmitted partially coherent flux is 0.069% of the total emitted flux, thus about 17% higher than for the 2D focusing case. The coherent flux transmitted through an aperture *l*_*x*_ × *l*_*y*_ at the lens position in Table 1 ▸ is 0.063% of the total emitted flux, as computed with the XUS undulator simulation tool in *XOP*. The optical simulations are performed with wider horizontal slits than the *XOP* estimate, but they include geometrical and reflectivity losses so the transmitted flux are similar to the *XOP* estimate.

## 4. Experimental method   

The experiment was performed on beamline 8-ID-I of the APS. Two 33 mm-period and 2.4 m-long undulator A provide bright coherent X-rays on 8-ID. They add in phase at 7.35 and 11 keV. Our test used only the upstream undulator A. Following the front-end, a small 0.275 mm-diameter water-cooled pinhole located 26.0 m from the source reduces the incident power on the beamline optics to a few Watts (Sandy *et al.*, 1999 ▸, 2010 ▸).

The white-beam slit is located downstream of the pinhole. Its opening can be estimated from equation (2), with *R*_l_ = 66.9 m, *R*_s_ = 27 m, λ = 1.127 Å (11 keV), and FWHM = 0.125 mm. The FWHM used here is a typical slit opening we use in front of the lens, and is significantly smaller than the vertical transverse coherence length calculated from equation (1) and shown in Table 1 ▸. We roughly set the white-beam slit opening to 32 µm but it was challenging due to mechanical issues with the slit stages. The tungsten edges are polished to 0.2 µm. The white beam apertured by the slit is reflected by a water-cooled Si mirror with a 2.5 mrad angle of incidence. The slit reduces the illumination of the mirror to approximately 12% of its 11.0 cm length. A Ge (111) double-crystal monochromator diffracting vertically was set to 10.6 keV (Narayanan *et al.*, 2008 ▸). Although we planned to work at 11 keV, the best focus ended up at 10.6 keV.

The focal length was adjusted by scanning the energy of the undulator and monochromator. The focal spot size was estimated by measuring the speckle size from an aerogel using a well known spatial autocorrelation technique (Sandy *et al.*, 1999 ▸, 2010 ▸). We note that this technique was recently extended to characterize the focal spot size of CRL at the LCLS (Sikorski *et al.*, 2015 ▸). The technique is capable of measuring the focal spot size in 2D with single-shot sensitivity, thus enabling X-ray laser shot-to-shot focus size monitoring. The focal spot size was measured by the transmitted intensity with a PIN diode while scanning a knife-edge using a cleaved GaAs wafer. These are well known to have atomically flat edges convenient to probe a micrometre focal spot, or to be used as slit blades (Dufresne *et al.*, 2009 ▸). The X-ray flux reported in this paper was calculated from the measured photocurrent from the diode.

## 5. Results   

**Figure 7:**
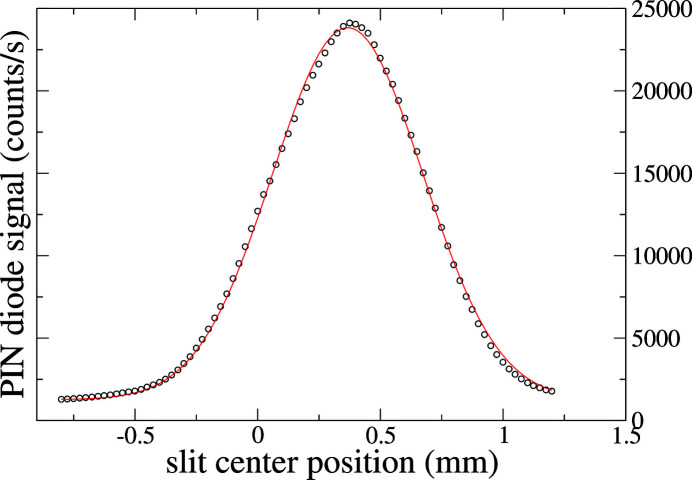
Horizontal beam profile in 8-ID-I just before the lens, with a white-beam slit opening of approximately 32 µm. The solid line is a Gaussian least-squares fit described in the text.

The horizontal beam profile in 8-ID-I upstream of the lens is shown in Fig. 7 ▸. It is measured with a 20 µm horizontal lens entrance slit placed 0.584 m upstream of the lens (see Fig. 1 ▸). It is much larger than the diffraction pattern of the white-beam slit because the white-beam slit acts as a pinhole camera as shown in Fig. 1 ▸. The measured FWHM of Fig. 7 ▸ is 0.757 mm, while using equation (8) one finds a FWHM of 0.754 mm, in excellent agreement with the data. A Gaussian least-squares fit is shown with a FWHM of 0.72 mm, and a small background of 1270 counts s^−1^. We note also that the measured beam profile is slightly asymmetric which we attribute to the fact that the two blades of the white-beam slits do not reside in the same plane. This effect has been shown to create an angle-dependent opening in coherence slits (Libbert *et al.*, 1997 ▸).

We chose the number of lenses *N* such that it would focus 11 keV X-rays in the usual 8-ID-I sample location. To ensure we achieved the best focus, we illuminated an aerogel sample with focused light and measured the speckle contrast and speckle size from a well known technique, discussed in Appendix *B* [App appb] and by Sandy *et al.* (2010 ▸).

**Figure 8:**
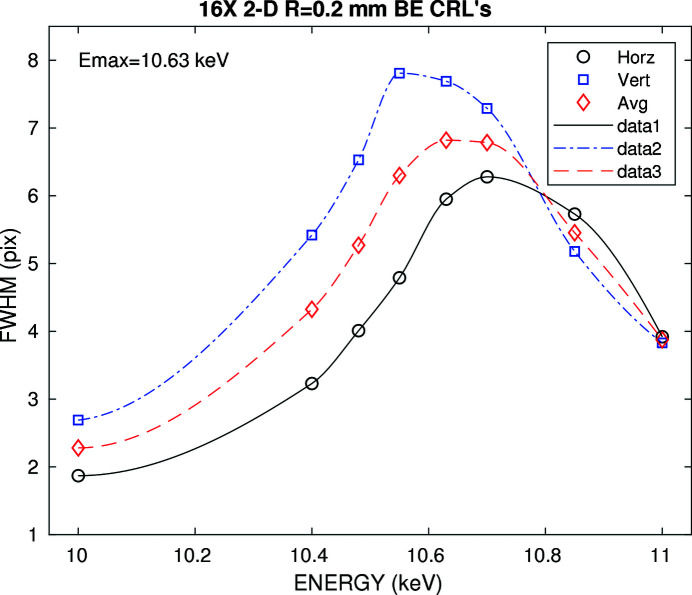
Measured horizontal and vertical speckle size from an aerogel pattern located in the focal plane. A pixel is 20 µm.

Fig. 8 ▸ shows the speckle size as a function of the incident X-ray energy in the horizontal and vertical direction. Since the speckle size is proportional to the width of the Fraunhofer diffraction pattern of the illuminated sample area, the speckle size should be maximum at the best focus energy. We note that the speckle size is larger in the vertical direction most likely due to the smaller vertical source size. The data also suggest a slight difference in focal length for the horizontal and vertical direction due to the difference in peak energy of the speckle size. Since *f* increases with energy, the horizontal speckle size peak maximum suggests the horizontal focus is upstream of the vertical one, in contrast with the simulations.

It is possible that this effect is caused by slope errors on the water-cooled Ge(111) monochromator. We have found that for vertical focusing we need to add more lenses to focus at a given distance (Zhang *et al.*, 2016 ▸). Zhang *et al.* vertically focused a 7.35 keV X-ray beam in 8-ID-I using the same geometry with Be lenses with *R* = 0.2 mm using *N* = 10 instead of *N* = 7, the best choice from optical constants and equation (11) (Zhang *et al.*, 2016 ▸). Calculations of this effect have been discussed recently (Antimonov *et al.*, 2016 ▸).

**Figure 9:**
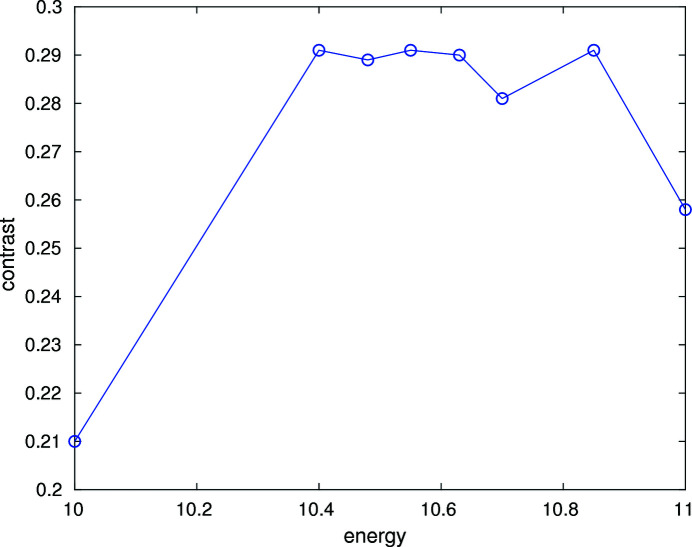
Measured speckle contrast from an aerogel pattern located in the focal plane.

Fig. 9 ▸ shows the speckle contrast as a function of incident energy. The contrast is maximum near 10.6 keV, but is not as sensitive to energy as the speckle size, and some small scatter is present near the peak energy. The maximum contrast is approximately 29%, which is close to the typical contrast on 8-ID-I in the unfocused condition.

In this experiment, we scanned the incident energy and doing so moved the focus longitudinally along the beam propagation direction. Given the quadratic energy dependence of δ(*E*) ∝ 1/*E* ^2^, a 100 eV shift reduction moved the focus upstream by Δ*z* = 2*f*Δ*E*/*E* ≃ 4 cm. It thus increased the illumination spot size by Δ_l_/*f*Δ*z* = 2Δ*E*/*E*Δ_l_ ≃ 2.3 µm. Thus we can also control the illuminated area by detuning the energy from the optimal focus. Changing the numerical aperture would also control the focal spot size.

**Figure 10:**
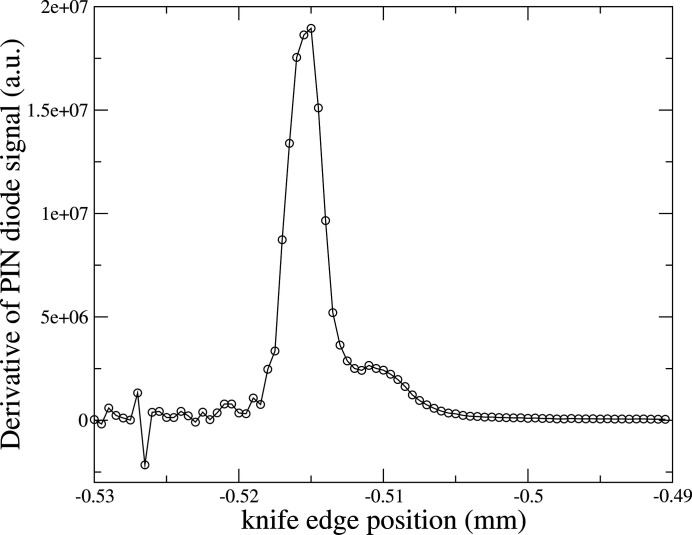
Vertical beam profile in the focal plane.

We chose to probe the focus with a knife-edge scan at 10.6 keV. Fig. 10 ▸ shows the results of the vertical knife-edge scan with a step size of 0.5 µm, after taking its spatial derivative. The vertical beam profile derived from the derivative has a FWHM of approximately 3 µm, and compares well with the simulations (2 µm). The vertical profile shows a long asymmetric tail with a possible diffraction fringe from the 125 µm slit. The fringe could be due in part to the increased transverse coherence in the vertical direction. Vertical fringes are visible in the simulation in Fig. 2 ▸.

**Figure 11:**
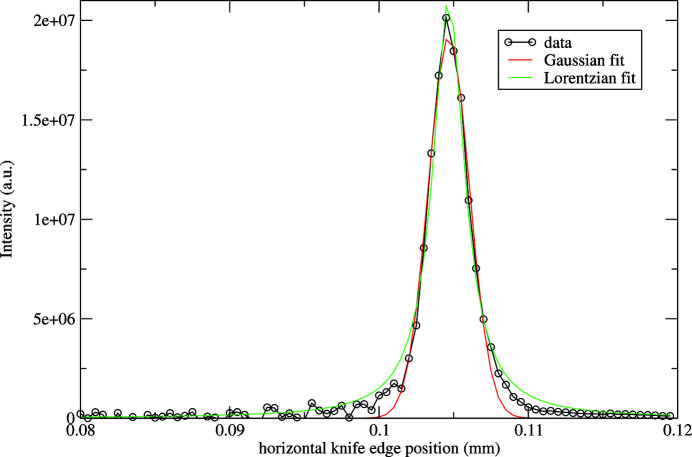
Horizontal beam profile in the focal plane from the derivative of the knife-edge scan, with a Gaussian and Lorentzian fit.

Fig. 11 ▸ shows the focal spot size with a Gaussian and Lorentzian non-linear least-squared fit. The FWHM of the data is 3 µm. It compares well with estimates made earlier of 2.9 µm. The data overall lie between the two line shapes, indicating some tails with larger background than a Gaussian line shape. We note that the horizontal profile is significantly smaller than the FWHM of the demagnified source size 2.35σ_*x*_(*R*_e_ − *R*_l_)/*R*_l_ = 22 µm. We achieved diffraction-limited focusing in the horizontal as well as in the vertical direction. This is in good qualitative agreement with the simulations performed.

The results were recently reproduced simultaneously on two beamlines 8-ID-E and 8-ID-I at the APS at 7.35 keV. The experimental conditions are shown in Table 2 ▸. For this experiment, both our inline undulators were closed to produce optimal flux at 7.35 keV. From equation (1), the vertical transverse coherence length at this energy is 318 µm in 8-ID-I, but we typically use 150 µm aperture before the lens when we focus vertically, thus using equation (2) with *l*_*y*_ = 150 µm, we set the white-beam slit to 40 µm horizontally, optimal for the 8-ID-I lens distance. A Si(220) crystal bisected the beam diffracted by the white-beam slit diffracting half of the beam in 8-ID-E while the other half continued to 8-ID-I and reflected from the horizontally deflecting Ge(111) double-crystal monochromator. We recently reported how we changed the 8-ID-I monochromator from vertical to horizontal deflection (Kearney *et al.*, 2018 ▸). The monochromator chamber was designed to accommodate either scattering direction (Narayanan *et al.*, 2008 ▸). The speckle contrast at high angles in 8-ID-E was measured from a single crystal of Fe_3_Al at its antiphase domain superlattice peak with a wavevector of 1.89 Å^−1^ (Brauer *et al.*, 1995 ▸). In 8-ID-E, a PI LCX:1300 CCD camera with 20 µm pixels was placed 1.1 m from the focal spot. Because the data in WA-XPCS have a diffuse peak with often arbitrary shape, the data were smoothed with a digital filtering technique developed earlier (Fluerasu *et al.*, 2005 ▸). In this test, the 8-ID-I camera is a Medipix 3 Lambda 750k PAD from XSpectrum with 55 µm pixels, placed 3.93 m downstream of the aerogel sample discussed earlier. The image distances *i* are for the horizontal and vertical direction, respectively. It uses the thin lens formula with the source at the white-beam slit position in the horizontal direction as stated earlier in Section 3. The calculated focal spot size *w* is from equation (12). We found that we needed to reduce the horizontal beam acceptance to 100 µm in 8-ID-I to produce the best focus. It is not clear why at this time. The results from the tests are shown in Table 2 ▸. The focal spot sizes compare well with the simple estimates provided in equation (12), particularly after correcting for the broadening from astigmatism using equation (14). It is not clear why the measured focal vertical spot in 8-ID-E differs from equation (14). We succeeded in focusing in 2D with round parabolic lenses on two beamlines, delivering good partially coherent flux and speckle contrast for XPCS experiments. This optical configuration was used for the remainder of 2019 at 7.35 keV for 25% of the beam time on 8-ID, with a partially coherent flux on the sample in 8-ID-E of 1.0 ± 0.5 × 10^10^ photons s^−1^, with the observed variation caused by different upstream alignment conditions.

More recently we also tested the same optics in 8-ID-E with the same conditions as Table 2 ▸ but with a wide-open horizontal white-beam slit (see Appendix *C* [App appc]). The condition results in a 2.8 µm vertical spot size consistent with Table 2 ▸, but the horizontal spot size is broader than the calculated image of the source or pinhole by 43 to 72%, respectively, which further adds evidence of a heat bump also present on the water-cooled Si monochromator.

## 6. Discussion   

Experimenters performing XPCS experiments typically define coherent flux as the partially coherent flux incident on their sample after an entrance aperture and some optics that produced speckle with a measurable contrast. The entrance aperture can be set to one coherent length or many, depending on the need for the experiment (Falus *et al.*, 2006 ▸). For samples that are radiation sensitive, one prefers to illuminate the sample with a single transverse coherent mode to maximize the contrast (DeCaro *et al.*, 2013 ▸).

Furthermore, the speckle contrast is maximum when a detector oversamples the speckle pattern, and starts to decline when the ratio of the speckle area to the pixel area here called *r*_s_ is less than unity (Dufresne *et al.*, 2002 ▸; Falus *et al.*, 2006 ▸). In the visible-light photon correlation spectroscopy community, it is well known that the signal-to-noise ratio of the time correlation function improves in fact and peaks at about *r*_s_ ≃ 0.1 for single speckle measurements (Schätzel, 1990 ▸). This scheme can be employed for samples that are insensitive to radiation damage (Dufresne *et al.*, 2002 ▸). For samples that are radiation sensitive, one finds it best to match the speckle area to the pixel area, both to enhance the range of wavevector covered by a camera and to maximize the contrast which is a parameter in the signal-to-noise ratio (Falus *et al.*, 2006 ▸; Vodnala *et al.*, 2018 ▸).

Returning to our measurements performed near 11 keV, we measured a focused partially coherent flux of about 1.3 × 10^10^ photons s^−1^. This is a factor 30% higher than without lenses in a 20 µm × 20 µm area and with a fully opened horizontal white-beam slit. Since 20 µm is a typical coherence slit opening used at 7.35 keV, we should compare the flux measured in an area (11/7.35)^2^ = 2.2 times smaller since the coherence lengths shrink by the ratio of energies. The partially coherent flux is thus about a factor 1.3 × 2.2 = 2.9 times higher than in unfocused conditions optimized for 11 keV. As noted in Table 1 ▸, the vertical opening in front of the lens could have been increased also to match the transverse coherence length in this direction (factor 1.8 possible). These tests were also performed with a single undulator A, but two inline phased undulators are available on the 8-ID straight section (factor 2.5 possible at 11 keV).

There is another valuable function of reducing the white-beam slit opening in conditions where the optics cannot preserve the brightness of the source due to spatial strain induced by the absorbed power density on the surface of the optics (Antimonov *et al.*, 2016 ▸). We reduced the total power on the first Ge(111) crystal of the double-crystal monochromator in 8-ID-I by a factor of seven with the smaller slit and were able to produce a diffraction-limited spot in the horizontal direction, and a much improved vertical focus on 8-ID-I. We note that these measurements were performed when the monochromator diffracted vertically (Kearney *et al.*, 2018 ▸).

For samples that are not prone to radiation damage, 2D focusing may be an advantage to match the speckle size to the pixel size. The PI camera used in this work has a pixel size of 20 µm, thus we measured a speckle size of around 120−140 µm, 4 m from the sample. This is fairly well matched to a modern PAD with pixel size ranging from 50 to 200 µm. Modern PADs absorb X-rays far more efficiently in their thick high-*Z* sensor (0.5 mm) than deep depletion silicon CCDs do, thus focusing may be helpful also with a PAD to allow their use with moderate sample–detector distance in existing facilities at X-ray energies above 10 keV.

We did not explore thoroughly the reduction of parasitic scattering from slits, Be lens and windows in this setup. A single 2D slit before the lens produced the SAXS speckle patterns of the aerogel sample. We found this setup remarkably free from parasitic scattering. In an ultra-small-angle scattering experiment, we have noted some background from the larger incoming angular divergence, thus one uses guard slits to reduce the long tails on the focus. Although, since 2015, 2D focusing has only been used at 7.35 keV, one should be able to image the pinhole described earlier and placed upstream of the white-beam slit to produce higher intensity on samples at 11 keV with some reduction in coherence. In light of recent evidence presented in Appendix *C* [App appc], one may require additional horizontal focusing to compensate for the heat bump of the Ge monochromator in 8-ID-I.

We note that this optical scheme could be helpful at other synchrotrons with horizontal source sizes comparable with or larger than the APS. Its use may also be helpful at high-brightness rings such as Petra III, NSLS-2, or the new multiple bend achromat sources (Tavares *et al.*, 2018 ▸) when focusing high-energy X-rays above 20 keV. A high-precision and stable slit is needed for this application.

## 7. Conclusions   

This paper demonstrated the use of a white-beam slit to spatially filter the horizontal coherence and focus the beam in two dimensions with refractive lenses, producing improvements in partially coherent flux and higher focal plane intensities on samples. Our water-cooled optics also benefit from the power reduction on the monochromators. The scheme is in routine use at two 8-ID beamlines sharing a single in-line straight section where two simultaneous XPCS experiments can be performed.

## Figures and Tables

**Figure 12:**
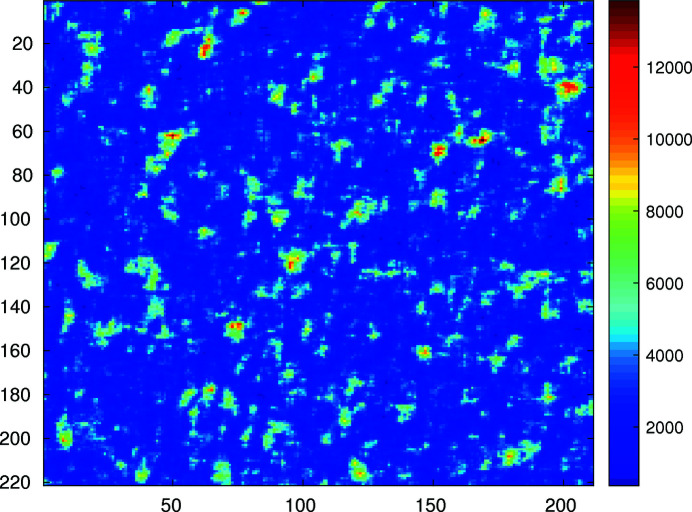
2D speckle pattern of the aerogel measured at 10.63 keV, with a PI camera with 20 µm pixels, 4 m from the sample. The color scale is displayed in analog-to-digital units for this 16-bit camera.

**Figure 13:**
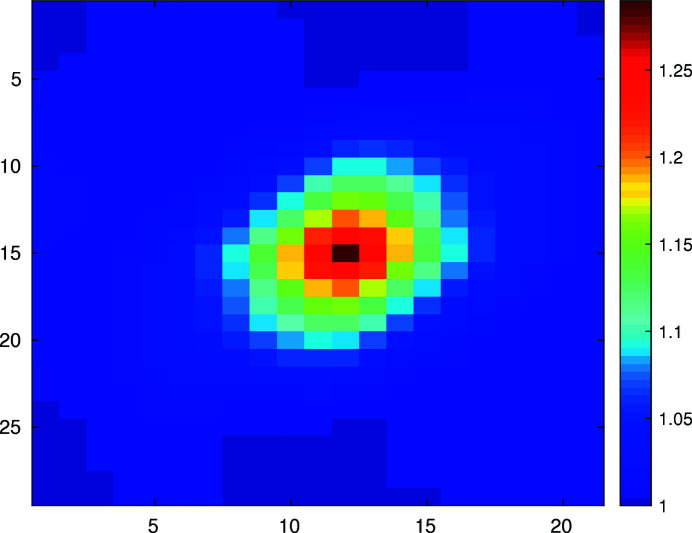
2D spatial autocorrelation function of a speckle pattern measured at 10.63 keV. The horizontal and vertical scales are in units of pixels.

**Figure 14:**
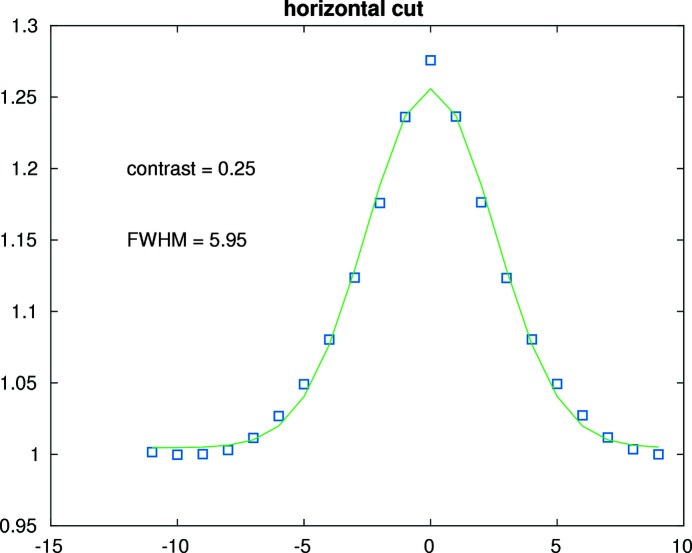
An horizontal slice of the 2D spatial autocorrelation function in Fig. 13. The solid line is a least-square fit to a Gaussian. The horizontal axis is in units of pixels.

**Figure 15:**
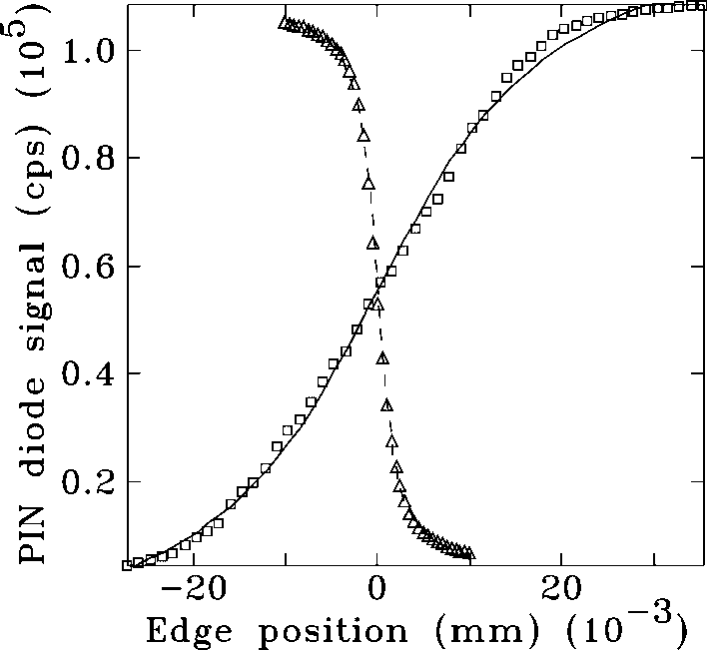
Horizontal (squares) and vertical (triangles) knife-edge scans of the beam profile in 8-ID-E. The solid and dashed lines are least-squares fit discussed in the text.

**Table 1:** Experimental parameters

X-ray energy	*E*	10.6 keV
RMS electron beam horizontal source size	σ_e*x*_	0.275 mm
RMS electron beam horizontal source divergence	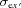	12.6 µrad
RMS electron beam vertical source size	σ_e*y*_	0.010 mm
RMS electron beam vertical source divergence	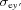	3.3 µrad
Horizontal white-beam slit opening	Δ	∼32 µm
Source to white-beam slit distance	*R*_s_	27.0 m
2D lens location	*R*_l_	66.9 m from source
Source to experiment distance	*R*_e_	69.2 m
Vertical transverse coherence length at lens	*l*_*y*_	207 µm
Horizontal transverse coherence length at lens	*l*_*x*_	8 µm
Horizontal transverse coherence length at white-beam slit	*l*_*x*_	3.3 µm
Be lens radius of curvature	*R*	0.2 mm
Number of Be lenslet	*N*	16
Dead layer of lenslet	*d*_0_	∼50 µm
2D aperture upstream of lens	(Δ_l_)^2^	0.125 mm by 0.125 mm
Sample to lens distance	*R*_e_ − *R*_l_	2.29 m

**Table 2:** Summary of the January 2019 results at 7.35 keV For 8-ID-I, *N* = 3.5 because we used three lenses with *R* = 0.1 mm and one lens with *R* = 0.2 mm. The ideal image distances *i* are nearly all larger than the sample-to-lens distance *R*_e_ − *R*_l_. The out-of-focus beam waist estimates *W*_H_ and *W*_V_ come from equation (14).

Beamline	8-ID-E	8-ID-I
Lens slit (H × V) (µm)	150 × 150	100 × 150
*R*_e_ − *R*_l_ (m)	1.645	2.29
*R* (mm)	0.1	0.1
*N*	5	3.5
*E* (keV)	7.35	7.35
*f* (m)	1.583	2.261
*R*_l_ (m)	53.2	66.9
*i* (m) (H × V)	1.685 × 1.631	2.40 × 2.34
*w*_H_/*w*_V_ (µm)	3.0 × 1.8	4.1 × 2.4
*W*_H_/*W*_V_ (µm)	4.7 × 2.2	6.3 × 4.1
Measured focal spot size (H × V) FWHM (µm)	5.0 × 4.5	4.7 × 3.7
Area detector resolution (µrad)	18	14
Speckle contrast, β	0.18	0.3
Partially coherent flux (photons s^−1^)	5.5 × 10^9^	1.5 × 10^10^
Horizontal monochromator	Si(220) single bounce	Double-bounce Ge(111)

## Appendix A Horizontal beam profile at the lens with a small white-beam slit opening

If we assume that the irradiance from a horizontal infinitesimal portion of the source at position and angular emission angle () is Gaussian in both spatial and angular coordinates, then the intensity generated by this element is

When we close the white-beam slit to an opening Δ, we image the source with a pinhole camera. Ignoring diffraction effects, the intensity at position *x*_l_ in the lens plane is an image of the source irradiance with

Replacing , and  in equation (16), we find that the beam profile in the plane of the lens is

and thus a Gaussian in coordinate *x*_l_ depending on both RMS source size and divergence, with a RMS beam size given in equation (8).

## Appendix B Speckle metrology

A typical pattern from an aerogel measured with the PI camera is shown in Fig. 12 ▸. The intensity is displayed with a color map from blue (low intensity) to red (highest intensity).

A standard technique to estimate the speckle size computes the 2D spatial autocorrelation function of the speckle pattern (Abernathy *et al.*, 1998 ▸; Tsui *et al.*, 1998 ▸; Sandy *et al.*, 1999 ▸). To remove the well known contribution due to Poisson noise of the detector (Dufresne *et al.*, 1995 ▸), we perform a time average of a cross-correlation function of two speckle patterns followed in time by one exposure period. Here, 63 such cross-correlation functions were averaged, and the overall impact on these measurements reduces the peak by 0.01. The cross-correlation function data are normalized to unity at large spatial lag.

A typical spatial cross-correlation function is shown in Fig. 13 ▸. Cuts in both directions were fit to Gaussians. A horizontal cut of Fig. 13 ▸ is shown in Fig. 14 ▸ with its Gaussian fit. The contrast is the peak value of the autocorrelation function and is slightly higher than the value obtained from the speckle size fit shown as a legend. The resulting FWHM in both direction were plotted in Fig. 8 ▸.

## Appendix C Beam profiles in 8-ID-E with a wide open horizontal white-beam slit

In July 2020, we performed a focusing test with the same lenses in 8-ID-E with a wide open horizontal white-beam slit. The conditions were the same as Table 2 ▸. Fig. 15 ▸ shows two beam profiles probed with the knife-edge, the sharp focus is in the vertical, while the broad one is in the horizontal. The fits are arctan fits, the FWHMs quoted are from the derivative of the fits. The vertical beam FWHM derived from the fit is 2.8 µm. One would expect to either demagnify the horizontal source (20 µm FWHM) or the pinhole (16.6 µm FWHM). We observe 28.6 µm FWHM horizontally. We suspect this broadening of the focal spot is caused by the heat bump of the Si monochromator which is not observed in the vertical. The flux was 4.35 × 10^10^ photons s^−1^ in this mode, while the following week it was 7.5 × 10^9^ photons s^−1^ with the WB slit closed to 40 µm, a factor of 5.8 higher. The ratio of the pinhole and the WB slit opening is 6.9, thus the flux is 18% lower than expected. This condition would provide very low contrast for XPCS, *i.e.* over ten coherence mode horizontally. These measurements complement our initial observation in 8-ID-I.
